# Selenium and Mercury Interactions in Apex Predators from the Gulf of Trieste (Northern Adriatic Sea)

**DOI:** 10.3390/nu10030278

**Published:** 2018-02-28

**Authors:** Jadran Faganeli, Ingrid Falnoga, Milena Horvat, Katja Klun, Lovrenc Lipej, Darja Mazej

**Affiliations:** 1 Marine Biological Station, National Institute of Biology, Fornace, 41 6330 Piran, Slovenia; katja.klun@nib.si (K.K.); lovrenc.lipej@nib.si (L.L.); 2 Department Environmental Sciences, Jozef Stefan Institute, Jamova, 39 1000 Ljubljana, Slovenia; ingrid.falnoga@ijs.si (I.F.); milena.horvat@ijs.si (M.H.); darja.mazej@ijs.si (D.M.)

**Keywords:** rays, selenium, mercury, Hg/Se ratio, coastal sea, Northern Adriatic

## Abstract

Since the environmental levels of selenium (Se) can moderate the bioaccumulation and toxicity of mercury (Hg) in marine organisms, their interactions were studied in seawater, sediments, plankton and the benthic (Bull ray *Pteromylaeus bovinus*, Eagle ray *Myliobatis aquila*) and the pelagic (Pelagic stingray *Dasyiatis violacea*) rays, as apex predators in the Gulf of Trieste (Northern Adriatic Sea). Male and female rays showed no difference in the Se contents in muscle tissue. Pelagic species contained higher Se levels in muscle but slightly lower levels in the livers of both genders. The Hg/Se ratios in seawater dissolved and colloidal fractions, plankton and sediment were <0.5, while those in particulate matter were <1.3. In benthic ray species, a parallel increase in Se and Hg in muscle was observed, so that an increased in Hg (MeHg) bioaccumulation results in Se coaccumulation. The Hg/Se ratios (molar) in muscle and liver of pelagic and benthic rays were <1.4 and <0.7, respectively. The low levels of Hg in muscle and liver in all the ray species corresponded to low Hg/Se ratios and increases in muscle and liver to 1 at 7 µg/g, dry weight (dw) and 5 µg/g dw, respectively, i.e., about 1.6 µg/g wet weight (ww).

## 1. Introduction

It was proposed that selenium (Se) moderates the bioaccumulation and toxicity of inorganic and organic mercury (Hg) in marine organisms [1,2,3,4,5] and in a wide variety of animal species [6,7,8]. Irreversible inhibition of essential selenoenzyme activities appears to be the primary mechanism of Hg toxicity [9,10,11]. The Se–Hg interaction has been described as antagonistic [8] and also synergistic [12]. The antagonistic effects in fish is complex and thought to be based on the formation of biologically inert Me–Hg-selenol complexes [13], and selenoneine [14] that could promote the excretion and demethylation of methyl mercury (MeHg). Recently, in vivo demethylation of MeHg mediated by Se was demonstrated to proceed in fish kidney [15] and intestine [16] and consequently the bioaccumulation of MeHg would decrease.

The animal intake of Se is crucial when the Se/Hg molar ratio in animal’s diet or tissues approach or exceed 1 [7]. In this context, a Se/Hg molar ratio of 1 can play an important role and serve as an indicator of susceptibility to Hg exposure where a more detailed Se and Hg speciation analyses is unavailable [4]. Therefore, a simple Se/Hg (or Hg/Se) molar ratio can be a useful indicator for assessing Hg toxicity [3].

In this context, we studied the interactions between Se and Hg in seawater, plankton and sediment, and in benthic (Bull ray *Pteromylaeus bovinus*, Eagle ray *Myliobatis aquila* and Common stingray *Dasyiatis pastinaca*) and pelagic (Pelagic stingray *Dasyiatis violacea*) rays in the Gulf of Trieste (Northern Adriatic Sea) using Hg and chemical speciation data of previously analysed ray specimens [17]. Same specimens were also analysed for arsenic [18]. Rays, like their relatives sharks and cartilaginous fish in general, are long lived apex predators with no known natural enemies and are known to be sensitive to high levels of Hg contamination through their food webs. Hence, they are a valuable bioindicators of Hg marine environmental pollution [19]. Since humans consume ray species, the results are valuable in terms of human health impact. The Gulf of Trieste is a shallow marine basin, (maximum depth of 25 m) and one of the most severely Hg polluted area in the Mediterranean and worldwide due to the input of large quantities of Hg from the Soča/Isonzo River inflow [20]. Hg pollution is a consequence of nearly 500 years of mining activity in Idrija (NW Slovenia), the second largest Hg mine in the world. In addition, we compare our data with previously obtained Se and Hg concentrations and Hg/Se ratios mostly in benthic organisms, including sponges, molluscs, crabs and some fish, from the Gulf of Trieste [21,22].

## 2. Materials and Methods

### 2.1. Samples

**Figure 1:**
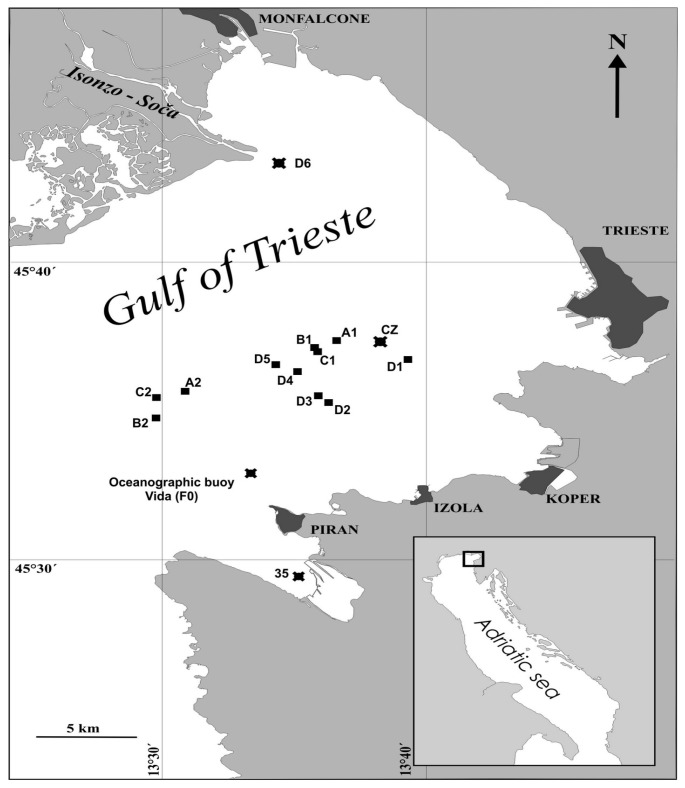
Map of the study area showing the sampling sites in the Gulf of Trieste (Northern Adriatic Sea), where ■ the four ray species and 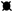 sediments were collected, and the oceanographic buoy Vida (sampling site F0) where the seawater and plankton samples were collected.

Seawater and plankton samples were collected at sampling site F0 at the Marine Biological Station oceanographic buoy VIDA (Figure 1). Seawater was collected at the surface (0.5 m deep) and at a depth of 20 m (1 m above the bottom) between June 2016 and May 2017. Samples were collected using acid pre-cleaned Teflon-lined Niskin samplers (10-L) and immediately decanted into acid cleaned 10-L polyethylene bottles. Seawater samples were filtered first through a 20 µm net and then through a 0.45 µm Millipore HA filters to discriminate between dissolved and particulate Se and Hg. To isolate colloids, the filtrates were additionally filtered through a 0.22 µm Millipore MCE filters and successively ultrafiltered through a membranes with a nominal molecular weight cut-off (MWCO) of 5 kDa using six Vivascience Vivaflow 200 units (Sartorius) with three Masterflex S/L membrane pumps (Cole-Palmer) at a flow rate of 300 mL min^‒1^ at 2.5 bar at 20 °C. The average concentration factor (CF) was 65. Plankton samples were collected using a 20 µm plankton net. The samples obtained using vertical net tows were fractionated at the laboratory through 200 µm and 50 µm nets to discriminate between mesoplankton and microplankton. All the particulate matter and plankton samples were rinsed with small volume of distilled water to remove salt and then freeze-dried.

Surface sediment samples in the Gulf of Trieste were collected at the sampling sites D6, 35, F0 and CZ (Figure 1) in December 2014 using a light gravity core sampler. Sediment cores (6 cm in diameter) were extruded and subsampled at 1 cm intervals to the depth of 20 cm. Samples were freeze-dried and ground to a fine powder for analysis.

In total, 17 bull rays (*P. bovinus*), five common eagle rays (*M. aquila*), both bottom dwelling benthic species from the family Myliobatidae, eight pelagic stingrays (*D. violacea*) representing pelagic species from the family Gasyatidae and 1 common stingray (*D. pastinaca*), were fished in the Gulf of Trieste (Figure 1) between September and October 2005. The biological data for these species is available in the literature [23,24,25]. Table 1 presents a summary of the biometric data for the four studied ray species. The bull ray is generally a benthic dweller, but can also swim in surface waters and can live up to 14 years. The common eagle ray (*M. Aquila*) is distributed throughout the eastern Atlantic as well as the Mediterranean. It inhabits shallow lagoons, bays and estuaries and it can be found offshore. Both species prey on bottom dwelling benthic invertebrates like crustaceans, molluscs, polychaetes and small benthic fish. The pelagic stingray *P. violacea* and the common stingray *D. Pastinaca* are the only member of stingray family (Dasyatidae) and belongs to the Elasmobranchii. The pelagic stingray has a worldwide distribution in waters warmer than 19 °C and is the only stingray that inhabits the open ocean. This species is typically found in surface waters down to a depth of 100 m, and its occurrence in the shallow Gulf of Trieste can be the result of tropicalization, i.e., the northward spreading of the southern species [26]. Since it is a pelagic dwelling predator, it is an active hunter preying on squids and pelagic bony fish. It has life span of 10 to 12 years. The Common stingray *D. Pastinaca* is a benthic predator in nearshore soft-bottom habitats.

Samples of liver and muscle were taken from each fish and kept frozen (−15 °C to −20 °C) until freeze-drying (Christ Alpha 1–4 Freeze–dryer, −40 °C, 0.020 mbar). The dry weight was then determined and the samples homogenised in a planetary mill (Fritsch planetary micro mill, Pulverisette 7) and refrigerated until analysis.

### 2.2. Analyses

*Filtered seawater samples* were diluted five times and Se measured by ICPMS (7700, Agilent, Santa Clara, CA, USA). Accuracy was checked by the use of reference material NRC-CNRC CASS-5 (Nearshore seawater reference material for trace metals). Limit of detection (LOD) was 0.1 ng/mL. Total dissolved Hg was determined, after BrCl digestion under UV light, BrCl inactivation with hydroxyl ammonium hydrochloride and reduction by SnCl_2_ [27], by cold vapour atomic absorption spectrometry (CV AAS, Milton Roy, Warminster, PA, USA). LOD was 0.1 ng/L.

*Samples of plankton and particulate matter* were digested together with filter (0.1 g) in Teflon tubes with the addition of 2 mL HNO_3_ (65%, suprapur, Merck) and 20 µL HF (40%, suprapur, Merck, Darmstadt, Germany) by microwave system (ULTRAWAVE, Single Reaction Chamber Microwave Digestion System, MILESTONE Inc., Shelton, CT, USA) at max power 1500 W: ramp to 240 °C 20 min and hold 15 min. The same procedure was applied for blank samples and reference material. The solutions obtained were diluted and measured by ICP-MS (7500ce, Agilent, Tokyo, Japan) equipped with an ASX-510 Autosampler (Cetac, Omaha, NE, USA). Instrumental conditions were: Babington nebuliser, Scott-type spray chamber, spray chamber temperature 5 °C, plasma gas flow rate 15 L/min, carrier gas flow rate 0.8 mL/min, make-up gas flow rate 0.1 L/min, sample solution uptake flow rate 1 mL/min, RF power 1500 W, reaction cell gases (hydrogen or helium) 4 mL/min, isotopes monitored ^78^Se and ^202^Hg. To check the accuracy of the results certified reference material BCR 414 Plankton was used. LOD for Se was 1 ng/g and for Hg 0.06 ng/g.

*Sediment samples* were digested with the use of HNO_3_/HCl/HF/H_2_O_2_ in closed teflon tubes in Microwave oven (Ethos1, MILESTONE) according to the procedure described in Vidmar et al., 2017. Digested solutions were measured by ICP-MS (7500ce, Agilent, Tokyo, Japan). Accuracy was checked by the use of reference material IAEA 433 (Trace elements and methylmercury in marine sediment). LOD for Se was 250 ng/g and for Hg 160 ng/g.

*Liver and muscle in rays* Se was determined within the multielemental analysis, preocedure for digestion and measurement are described in Šlejkovec et al. [18]. LOD for Se was 15 ng/g. Procedure for Hg was described in Horvat et al. [17].

## 3. Results and Discussion

### 3.1. Seawater

Seawater at F0 contained 0.32–2.13 µg/L of dissolved Se, of which about 12% was in the colloidal (>5 kDa) form (Table 2) in accordance with previously reported data from the gulf [28]. In the suspended particulate matter (SPM, 0.45–20 µm), Se concentrations averaged 0.01 µg/L (0.16 µg/g, dry weight, dw) originating mostly from the input of particles from the Isonzo/Soča River inflow, the main tributary entering the gulf. Picoplankton (0.45–2 µm) in the particulate matter is composed of mainly cyanobacteria and heterotrophic bacteria [29] whereas nanophytoplankton (2–20 µm) in the particulate matter is of mainly coccolitoforides and unidentified flagellates of several taxonomic groups. Silicoflagellates play only a minor role [30]. Levels of dissolved and particulate Se in seawater from the gulf are about 2–20 fold higher than in the open ocean [31].

Dissolved Hg at F0 was <1 ng/L and nearly all colloidal (Table 2). Particulate Hg averaged about 1 ng/L. Higher levels of dissolved (up to 5 ng/L) and particulate (up to 64 ng/L) Hg are restricted to the surface water layer in the northern part of the gulf affected by the Soča/Isonzo River discharge [32].

### 3.2. Plankton

**Figure 2:**
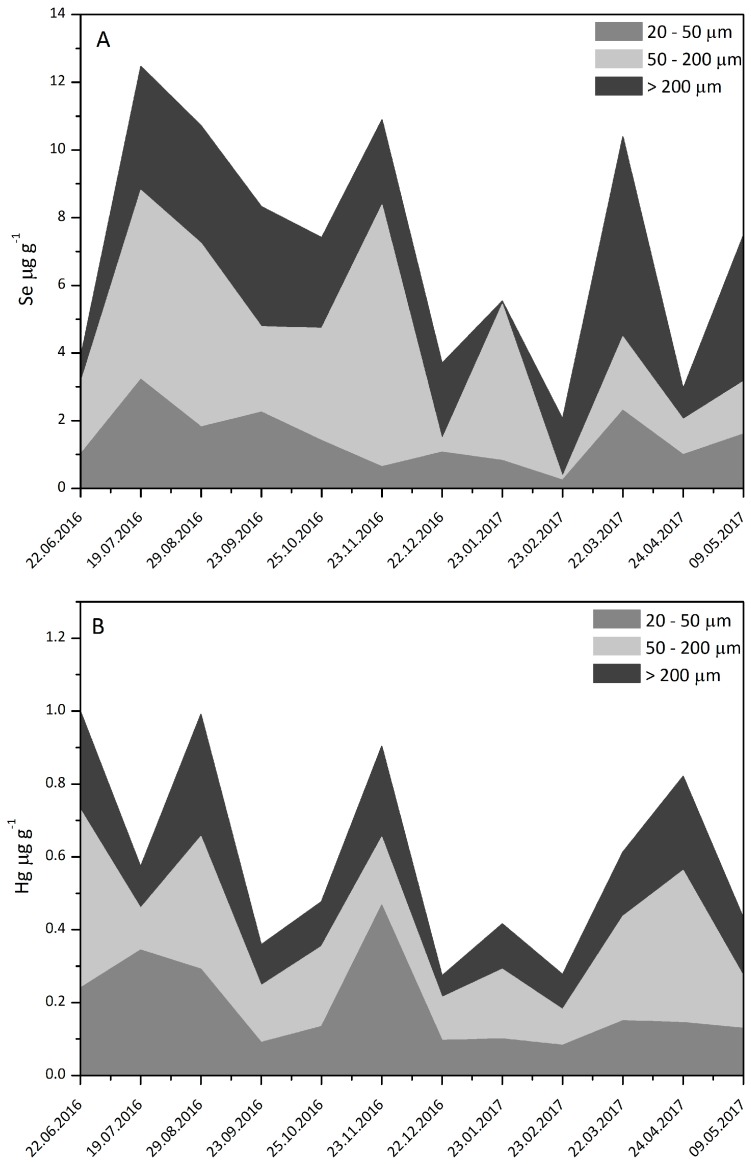
Temporal variation of Se (**A**) and Hg (**B**) concentrations in size-fractionated plankton in 2016–2017.

Meso(zoo)plankton (>200 µm) collected at F0 contains up to 5.9 µg/g dw of Se (Table 2) and lower winter Se levels (Figure 2) can be a consequence of a lower mesozooplankton biomass compared to summer and autumn [33]. In the plankton (Table 2), the highest levels of Se occur in the >200 µm mesoplankton (BDL—5.9 µg/g dw) and 20–50 µm microplankton (0.64–3.2 µg/g, dw) fractions and the lowest in the 50–200 µm microplankton (0.37–0.63 µg/g dw) fraction. This is probably related to the taxonomic composition and feeding behavior of the different species in the various plankton fractions. The 20–50 µm microplankton fraction is composed mostly of diatoms in spring and autumn, dinoflagellates in summer and nanoflagellates in winter [30]. Microzooplankton (50–200 µm) consists of mainly ciliates over the entire year and feed on nanoflagellates [30]. Mesozooplankton in spring, autumn and winter comprises mainly of copepods (*Acartia clausi*) while in summer the cladoceran (*Penilia avirostris*) and dinoflagellate such as *Noctiluca miliaris* are more abundant [30]. Copepods prey on diatoms and microzooplankton while cladocerans feed on other autotrophs and heterotrophs [33,34].

At F0, the levels of Hg (Table 2) in the 20–50 µm microplankton fraction are between 8–541 ng/g dw, between 211–422 ng/g dw in the 50–200 µm fraction and between 11 and 342 ng/g dw in the >200 µm mesoplankton fraction. These levels are comparable to those previously reported for the Gulf of Trieste [32].

### 3.3. Sediments

Concentrations of Se (Table 2) in the gulf’s surface sediments (0–1 cm) ranged from 0.28 to 2.01 µg/g and between 0.53–2.58 µg/g in deeper (10–20 cm) layers. The highest levels were in the central part of the gulf (sampling site CZ) where sediments are mostly composed of silty sands and this zone acts as a sediment depocentre [35]. Total Hg distribution (Table 2), as previously reported by Covelli et al. [36], is represented by a decreasing gradient from the area affected by the Soča/Isonzo River inflow (2.2 µg/g) out towards the southern part of the gulf (0.3–0.6 µg/g). Coarse sediments prevail along the northern littoral zone and grain-size decreases towards the southern part [35].

### 3.4. Fish

**Figure 3:**
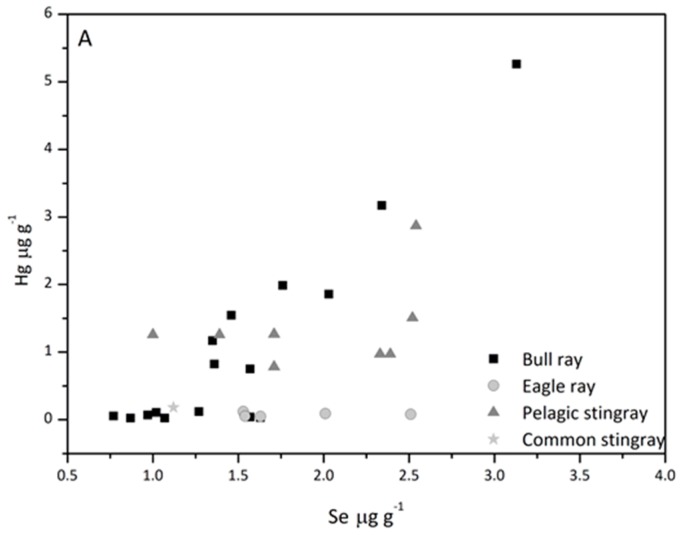

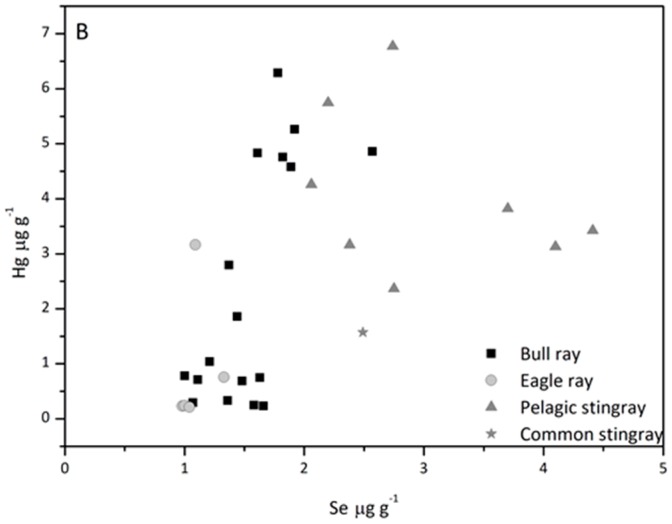
Se and Hg contents in liver (**A**) and muscle (**B**) of rays.

The Se contents in ray species (Figure 3) vary from 1.0–4.41 µg/g dw in male and female muscle with no differences between sex. The pelagic species contain slightly lower Se levels in the liver but the highest levels in the muscle (Figure 3), in conformity with high Se levels in plankton and seawater (Table 2). Significant correlations were found between Se contents in liver (*r*^2^ = 0.64) and muscle (*r*^2^ = 0.54), and weight in benthic rays, also considering that specimens of *P. bovinus* encompassed a wide weight/age spectrum (Table 1).

The highest levels of Hg (45–100% as MeHg) were present in the muscle of *D. violacea* (1.17–4.40 µg/g dw), followed by *P. bovinus* (0.13–3.05 µg/g dw), and *D. pastinaca* (0.07–0.47 µg/g dw) [17]. The contents of Hg were found to show a positive correlation with weight and age but in *P. bovinus* the more gradual increase in the level of MeHg was independent of Hg attributed to demethylation as part of a detoxification mechanism [17]. In benthic species, there is a similar increase of Se and Hg in muscle (Figure 3) and Hg (MeHg) bioaccumulation results in Se coaccumulation. High Se levels observed in the muscle of pelagic feeding species (Figure 3) are in accordance with previously estimated greater bioaccumulation of Hg (MeHg) by pelagic feeding species *D. violacea*, which first appeared in the Northern Adriatic in 1999, since this species might not be adapted to high Hg levels in the local marine environment [17].

A comparison with other mainly benthic organisms, such as *Pagurus* s.p., *Pilumnus* s.p. *and Portus depurator* which are frequently preyed by benthic rays, from the Gulf of Trieste have a Se content of 0.12–0.52 µg/wet weight, ww or ~0.48–2.08 µg/g dw [21,22]. Exceptions were mussels (*Mytilus galloprovincialis*) from the Hg contaminated northern part of the gulf (1.12 µg/k ww, ~4.5 µg/g dw). The Se content of the benthic dwelling fish red mullet (*Mullus barbatus*) muscle, caught in the Hg contaminated northern part of the gulf was on average 0.27 µg/g ww (~1.1 µg/g dw). The Se contents in the liver and kidney was 1.72 µg/g ww (~6.9 µg/g dw) and 1.56 µg/g ww (~6.2 µg/g dw), respectively, which is about 2–3 fold lower than in the less contaminated areas [22]. In benthic organisms, levels of Hg were from 0.01–0.34 µg/g ww (~0.04–1.4 µg/g dw) and those of mussels between 0.04–0.2 µg/g ww (~0.2–0.8 µg/g dw) and up to 1.6 µg/g ww (~6.4 µg/g dw) in the Isonzo/Soča polluted area of the gulf. The amounts of Hg in red mullet from the Hg uncontaminated areas of the gulf were 0.1. µg/g ww (~0.4 µg/g dw) in muscle and 0.2 µg/g ww (~0.8 µg/g dw) in liver and kidney but increased to 0.2 µg/g ww in muscle and 0.4 µg/g ww in the liver and kidney in the contaminated northern part of the gulf.

### 3.5. Hg/Se Ratio

The Hg/Se ratios (molar) (Table 2) in seawater dissolved phase was <0.01, 0.43 in the colloidal phase and <1.3 in the particulate phase. In plankton, Hg/Se ratios were <0.4 with higher ratios in microplankton compared to mesoplankton. The surface sediment had ratios of <0.4.

**Figure 4:**
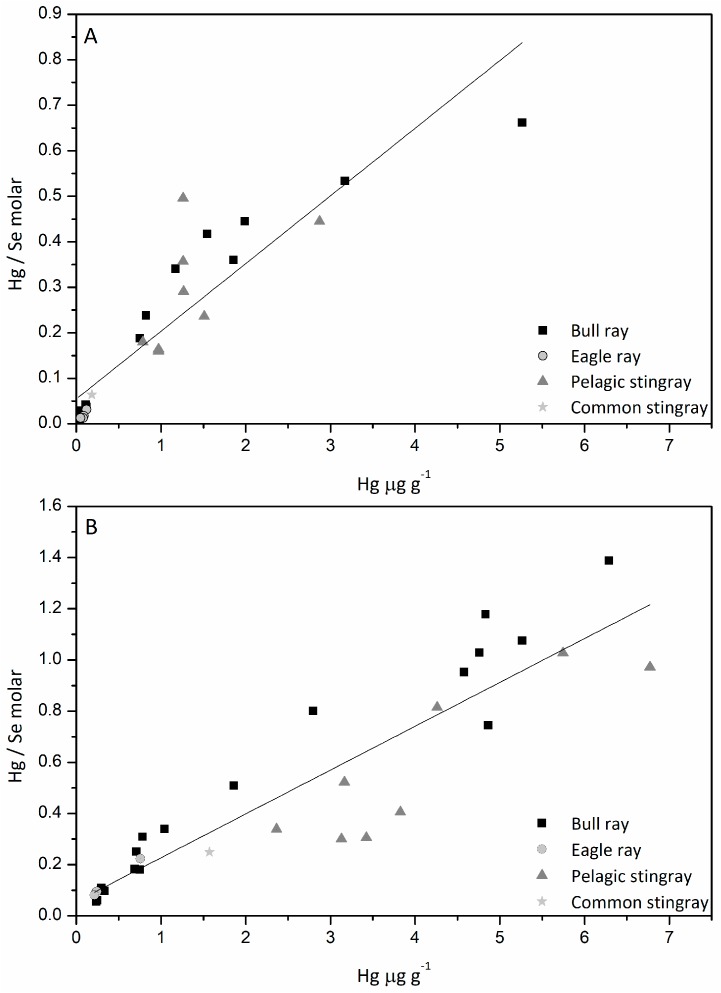
Relationship between Hg/Se ratios (molar) and Hg contents in liver (**A**) Hg/Se = 0.15Hg + 0.05 (*r*^2^ = 0.82, *n* = 29), and muscle (**B**) Hg/Se = 0.17Hg + 0.06 (*r*^2^ = 0.84, *n* = 30) of rays.

The Hg/Se ratios in muscle of pelagic and benthic ray species were <1.6 while in the liver it was <0.7 (Figure 4) with little differences between species. The Hg/Se ratios in liver (*r*^2^ = 0.64) and muscle (*r*^2^ = 0.72) of benthic rays were significantly correlated with weight of specimens. The low content of Hg in muscle and liver in all the ray species corresponds to a low Hg/Se ratios that increase to 1 in liver and muscle at a level of about 7 and 5 µg/g dw, respectively, corresponding approximately to 1.8 and 1.3 µg/g ww and exceeds the limit for Hg content (1 µg/g ww) in fast accumulating species including predators and benthic species reported in the EU food safety legislation [37]. Similarly, Storelli and Marcotrigiano [38] found that low Hg concentrations correspond to low Hg/Se ratios in the liver of the Mediterranean deep sea dwelling Blackmout catshark (*Galeus melastomus*), a relative of the ray, and that this ratio reaches 1 at Hg concentrations >1 µg/g ww. Hg/Se ratio of about 1 was found in the muscle of marbled electric ray (*Torpedo marmorata*), a species belonging to the same order as rays and fished in Kvarner (central Adriatic). It contained 0.26 µg/g f.w. (~1 µg/g dw) Se and 0.65 µg/g ww (~2.6 µg/g dw) Hg, respectively [21]. Comparison of Hg/Se ratios with other marine organism from the gulf is possible using previously published data on Hg and Se mostly from the analyses of benthic organisms and some fish [21,22]. The species analysed include *Mytilus galloprovincialis* and muscle tissue of the benthic fish *Mullus barbatus* from the northern part of the gulf and revealed Hg/Se ratio <0.6. Similar ratios were described in *Mullus barbatus* from the Northern Adriatic open waters [39].

## 4. Conclusions

The high Hg/Se ratios (molar) in seawater dissolved and colloidal fractions, plankton and surface sediment were <0.5 while those in particulate matter were <1.3 due to high Se contents in the Gulf of Trieste. In benthic ray species, higher Se contents and higher Hg/Se ratios in liver and muscle corresponded to higher weight (age) of specimens. Benthic rays exhibited a parallel increase of Se and Hg in muscle, so that increased Hg (MeHg) bioaccumulation results in Se coaccumulation. In pelagic species, high Se levels in muscle corresponded to greater bioaccumulation of Hg since this species might not be adapted to high Hg levels in the local marine environment. The Hg/Se ratios (molar) in muscle and liver of pelagic and benthic rays were <1.6 while in liver was <0.7. Low Hg contents in muscle and liver of all ray species corresponded to low Hg/Se ratios increasing in liver and muscle to 1 at a level of about 7 and 5 µg/g dw (~1.6 µg/g ww). It appears that Se in apex predators in the Gulf of Trieste, characterized by rather high seawater Se levels, moderates the bioaccumulation and environmental toxicity of Hg at levels higher than those set by EU food safety legislation.

## Figures and Tables

**Table 1:** Biometric data of the four studied ray species [17].

Species	*n* (m, f, juv)	Disc Width (mm)	Disc Length (mm)	Weight (kg)
Eagle ray (*Myliobatis aquila*)	5 (1, 1, 3)	273–380, 310 ± 50	142–225, 176 ± 38,9	0.26–0.98, 0.50 ± 1.31
Bull ray (*Pteromylaeus bovinus*)	17 (6, 9, 2)	450–2220, 727 ± 422	760–2940, 1714 ± 606	1.50–116.0, 47.0 ± 43.0
Pelagic stingray (*Dasyatis violacea*)	8 (5, 3, 0)	437–600, 531 ± 61	1010–1392, 1240 ± 146	2.40–7.56, 4.83 ± 1.78
Common stingray (*Dasyatis pastinaca*)	1 (1, 0, 0)	455	367	4.00

The numbers of males (m), females (f) and juveniles and embryos (juv) are given in parentheses.

**Table 2:** Summary of Se (µg/kg) and Hg (µg/kg) concentrations and Hg/Se (molar) ratios in the water column and surface sediments of the Gulf of Trieste.

	Se	Hg	Hg/Se
**Seawater dissolved**	1.58–2.37	BDL—5.03 10^‒3 a^	<0.01
**Seawater colloidal**			
(>5 kDa)	0.032–0.04	BDL—1.98 ^b^	0.43
**Seawater particulate**			
(0.45–20 µm)	158	BDL—4.35	<1.3
**Microplankton**			
20–50 µm	639.9–3223.2	8.0–540.7	0.01–0.22
50–200 µm	371.3–632.0	120.6–422.1	0.01–0.36
**Mesoplankton**			
(>200 µm)	BDL-5901.3	10.05–341.7	<0.12
**Sediment surface**			
(0–1 cm)	275.9–2599.1	110.5–2291.4	0.05–0.35

^a^ Faganeli et al. [29]; ^b^ Koron et al. [25]; BDL—below limit of detection (LOD).
